# Event and Comment

**Published:** 1922-07

**Authors:** 


### DENTAL REGISTER

THE MAGAZINE OF DENTISTRY

Vol. LXXVI JULY, 1922 No. 7

### EVENT AND COMMENT

**Partial Dentures.** The advent of bridgework made a distinct impression on the methods formerly employed to make denture structures by some form of plate construction practicable; and strange to say that, like many other good and bad things as well, much fault is still found with most forms and methods of construction, and we do not as yet see any new and better method in sight.

We have got to have bridges of various types, as our patients will not consent to the various and usual more or less unstable substitutes that seem possible by the usual forms or expedients as they are at present constructed.

We believe partial dentures can be made in place of most forms of bridges that will be serviceable and not be a menace to the adjacent tissues of the mouth, or seriously increase the probabilities of undesirable and unsanitary conditions of the mouth.

Probably the first and most important matter to be adjusted is their technical or mechanical adaptations, making such appliances as stable as such demands can be properly constructed. Then they should be free from undue stress and other counter irritations of occlusal and articular interferences. If these matters are adequately and kindly adjusted there should be no unsurmountable difficulties why partial dentures may not be made and worn safely and comfortably. This is a problem, however, that the profession will have to study and work out more carefully than it has heretofore attempted.

The hygienic and functional requirements are so intricate and exacting that much greater ingenuity and wisdom will need to be expended in accomplishing this matter than the profession generally has thought necessary. The problem is imperative and it is clamoring for recognition; and our wisest and best men can not avoid giving it proper and needed attention.

If the profession really believes that this particular matter is of vital importance, and there seems a possible solution, we are sure we will tackle it with the usual energy and scientific purpose needed.

